# Incisional Hernia: A Surgical Complication or Medical Disease?

**DOI:** 10.7759/cureus.50568

**Published:** 2023-12-15

**Authors:** Islam Omar, Tilemachos Zaimis, Abby Townsend, Mohamed Ismaiel, Jeremy Wilson, Conor Magee

**Affiliations:** 1 General Surgery, The Hillingdon Hospitals NHS Foundation Trust, Uxbridge, GBR; 2 General Surgery, Wirral University Teaching Hospital NHS Foundation Trust, Wirral, GBR; 3 General Surgery, Altnagelvin Area Hospital, Londonderry, GBR

**Keywords:** abdomen ventral hernia, incisional ventral hernia, mesh repair, laparotomy, strangulation, bowel obstruction, emergency hernia, elective hernia, incisional hernia

## Abstract

Incisional hernia (IH) is a frequent complication following abdominal surgery. The development of IH could be more sophisticated than a simple anatomical failure of the abdominal wall. Reported IH incidence varies among studies. This review presented an overview of definitions, molecular basis, risk factors, incidence, clinical presentation, surgical techniques, postoperative care, cost, risk prediction tools, and proposed preventative measures. A literature search of PubMed was conducted to include high-quality studies on IH.

The incidence of IH depends on the primary surgical pathology, incision site and extent, associated medical comorbidities, and risk factors. The review highlighted inherent and modifiable risk factors. The disorganisation of the extracellular matrix, defective fibroblast functions, and ratio variations of different collagen types are implicated in molecular mechanisms. Elective repair of IH alleviates symptoms, prevents complications, and improves the quality of life (QOL). Recent studies introduced risk prediction tools to implement preventative measures, including suture line reinforcement or prophylactic mesh application in high-risk groups.

Elective repair improves QOL and prevents sinister outcomes associated with emergency IH repair. The watchful wait strategy should be reviewed, and options should be discussed thoroughly during patients' counselling. Risk stratification tools for predicting IH would help adopt prophylactic measures.

## Introduction and background

Incisional hernia (IH) is defined as an abdominal wall gap with or without a bulge at the site of a previous surgical scar detected by clinical examination or imaging [1].

Complex IH

There is no clear-cut definition for the term complex IH. However, it is used to describe IH with one or more of the following characteristics: a large hernial defect, enterocutaneous fistula, several hernias situated anatomically apart from each other, IH close-to-bone or associated with local infection, loss of domain (LOD), and re-recurrence [2].

Loss of domain

There is a lack of consensus on a precise definition of LOD in the existing literature. Clinically, it can be diagnosed when the herniated contents cannot be reduced below the fascial level in the supine position [3]. A more accurate modality to diagnose LOD is cross-sectional imaging, appreciating the ratio between the herniated and the intra-abdominal volumes. Some set the threshold at an extraperitoneal volume between 20 and 25% to diagnose LOD [4,5]. In comparison, others diagnose LOD if the extraperitoneal volume approaches 50% or more, i.e., when the ratio of hernia sac volume to the abdominal cavity volume is ≥ 0.5 [3].

## Review

Incidence

The reported incidence of IH in the current literature is quite variable, ranging from below 5% to as high as 70% in some series. This wide variation is attributed to the variation in the primary surgical pathology, surgical incision site and extent, associated medical comorbidities, and previous exposure to risk factors [6-8]. A systematic review of renal transplant patients showed an IH rate of 1.1% - 7% after an open renal transplant, with a mean of 3.2% [9]. A recent Sweden's Renal Cell Cancer Database study analysed 6417 patients to determine the comorbidities and subsequent development of IH. Of these 6,417 patients, 19% (1,201 individuals) underwent minimally invasive surgery, whereas 81% (5216 individuals) had open surgery. After a five-year follow-up period, the IH development rate was 2.4% (1.0-3.4%) following minimally invasive surgery and 5.2% (4.0-6.4%) after open surgery (p<0.05). In the open surgery group only, IH was significantly associated with left-sided surgery and age (both p<0.05) [10].

Conversely, a recent study that included 157 patients with abdominal aortic aneurysm (AAA) showed that IH incidence after open repair of AAA was 46.5%, with a median time for IH development of 24.43 months. The risk factors identified were active or previous smoking, chronic kidney disease, and previous abdominal surgery [11]. IH can develop in young people and even in infant populations undergoing abdominal surgery. A recent study from the Netherlands analysed 2055 infants under three years old who had abdominal surgery between 1998 and 2018. One hundred and seven infants (5.2%) developed IH. However, the incidence was variable among the different primary surgical pathologies; necrotising enterocolitis (12%), gastroschisis (19%), and omphalocele (17%) had the highest incidences of IH. Wound infection, preterm birth, and history of stoma were all identified as significant risk factors for developing IH [12]. The high rate of open surgery and the occurrence of IH through more minor abdominal defects after minimal access surgery, including both laparoscopic and robotic procedures, contribute to the high prevalence of IH [13]. A meta-analysis of 24 trials included 3490 patients to study the rates of IH after laparoscopic versus open abdominal surgery. The results showed that the incidence of IH was significantly lesser in the total laparoscopic procedures. However, laparoscopically assisted procedures did not significantly reduce IH compared to open surgery [14]. Despite that, port-site variant IH has been commonly reported [15].

Aetiology and molecular basis

Hernias that occur in the early postoperative stage result from inadequate closure and faulty surgical technique. In the presence of wound infection, the neutrophil’s local inflammatory response and proteolytic enzymes disrupt the normal wound healing process by interrupting collagen synthesis [16]. However, the late-onset development of IH points to the disorganisation of the extracellular matrix (ECM) and the disequilibrium of collagen metabolism when collagen breakdown exceeds synthesis [17-19].

A comparative study examined the hernial fascial ring tissue (HRT) and hernia sack tissue (HST) harvested from patients undergoing hernia surgery compared with normal fascia (FT) and peritoneum (PT) by histology and immunofluorescence. Compared to the control, there were alterations in tissue architecture, fibroblast morphology, and ECM organisation in the IH tissues. These findings support the heterogeneity of the fibroblast population at the laparotomy site that could contribute to the development of IH [20].

Collagen disorganisation and impaired fibroblast function compromise the abdominal wall's mechanical integrity, leading to IH [19,21]. These changes in mechanical properties initiate repair reactions within load-bearing tissues, like ligaments and tendons. Furthermore, the cells in tendons and ligaments that load-bear resist hypoxic and ischemic insults after injuries. Oppositely, muscles of the abdomen, when exposed to ischemia, induce fibroblasts to generate atypical collagen, leading to an impaired ECM [18,22].

During wound healing, platelets and fibrin produce a provisional matrix, serving as a transient scaffold that attracts other critical components for effective wound restoration. Insufficient haemostasis with the generation of haematoma can interrupt this ECM resulting in IH [23,24]. The temporary matrix draws in inflammatory cells and signalling molecules, initiating inflammatory classical pathways. When the inflammatory response is delayed or persists for a prolonged period, it culminates in the activation of pathogenic fibroblasts, ultimately causing disorganisation of the ECM [25].

The structural strength of connective tissues depends on the balance between collagen type I and type III. This is because the intermolecular bonds between collagen type I and type III contribute additional tissue strength [26,27]. Type I collagen is fibrous, strong, and thicker in diameter than type III collagen [28]. Tissues from IH patients showed a decreased collagen type I to type III ratio, resulting in a disorganised ECM [29,30].

Additionally, skin fibroblasts’ increased secretion of type III collagen has been linked to the onset of IH as collagen type III imparts weaker mechanical properties to the tissue [17]. On the other hand, fibroblasts play a pivotal role in ECM repair and wound healing. Another proposed mechanism for wound failure is the existence of abnormal fibroblasts secondary to reduced levels of growth factors or cell cycle arrest due to ischemia [17,31]. Xing et al. identified an atypical fibroblast population as the culprit behind the secretion of modified collagen phenotype in the early failure of laparotomy wounds [32]. Moreover, these neutrophils exhibit different chemotactic responses. Fibroblasts’ phenotype selection is influenced by the reduction in the abdominal wall’s mechanical strength [32].

Diaz et al. studied the changes in the fascia of IH patients [33]. A notable thinning of the ECM reduced fibroblast density, minimal presence of immune cells, and dysmorphic fibroblasts exhibiting limited interaction with the surrounding matrix were observed. IH tissue’s fibroblasts exhibited a spindle-like bipolar shape with a decreased surface area and demonstrated a more pronounced vimentin network than actin expression. Examination under electron microscopy unveiled cytoplasmic vacuolation and swelling of the mitochondria. In response to fibronectin and collagen type I, these fibroblasts exhibited enhanced proliferation, reduced adhesion, and quicker migration. Additionally, the fibroblast cells from the IH demonstrated heightened sensitivity to apoptosis and autophagy [33].

Proline hydroxylase and lysine hydroxylase catalyse collagen cross-linkage to enhance mechanical stability [34]. The structural tissues of IH patients show lower hydroxyproline content. Furthermore, fibroblasts cannot transport hydroxyproline in these patients, reducing cross-linking and enhancing collagen solubility. This condition ultimately leads to mechanical failure [35,36].

Understanding the molecular basis of IH pathogenesis would enable early prediction to adopt preventative measures. The degradation products of collagen are released into the bloodstream during tissue remodelling after injury or surgical trauma. These fragments are called neo-epitopes and can be considered serum biomarkers for collagen turnover [37-39].

Henriksen et al. observed a higher turnover of collagen type IV when compared to collagen type V in IH patients preoperatively [39]. The serum concentration of N-terminal pro-peptide of type IV collagen 7S domain (P4NP-7S), which is a breakdown product of collagen type IV, was observed to be increased in IH patients and is considered to be linked to the development of IH [40]. These results imply that collagen degradation products have diagnostic significance.

Moreover, alterations in the matrisome structure and the existence and growth of anomalous fibroblasts are causative factors in developing IH. The ischemia at the incision site induces the accumulation of truncated ECM, resulting in prolonged wound healing. Additionally, the changes in the quantities and proportions of various collagen types are the primary underlying factor for the disorganisation of the ECM. Neo-epitope measurement is a promising diagnostic tool [41].

Risk factors

IH is associated with a multitude of risk factors, encompassing male gender, smoking, and comorbidities (such as diabetes mellitus (DM), chronic obstructive pulmonary disease (COPD), and obesity). Furthermore, hypoalbuminemia, immunosuppression (e.g., via steroids and chemotherapy), exposure to radiotherapy, malignancy, connective tissue disorders, operative-related factors, and postoperative complications (e.g., intra-abdominal collections and abdominal sepsis) constitute additional risk factors [2].

A recent study examined the molecular mechanisms of IH and the association of these factors with smoking, abdominal aortic aneurysms, obesity, diabetes mellitus, and diverticulitis [42]. The results showed that the levels of collagen I and III, matrix metalloproteinases, and tissue inhibitors of metalloproteases are abnormal in ECM of IH patients, and ECM disorganisation has overlapped with these comorbid conditions. This could partly explain the association of IH with these comorbidities. Moreover, BMI is a known risk factor for local wound complications after surgery, which can eventually compromise the healing process and lead to IH [43,44].

A Swedish study included 1,621 patients with vascular procedures and laparotomies for bowel procedures in 2010 [45]. They revealed that wound infection posed a risk factor for wound dehiscence and IH. Moreover, an elevated BMI (exceeding 30 kg/m^2^) was recognised as a risk factor for wound dehiscence [45].

The same set of risk factors has been proven to be associated with IH, and the results have been reproduced through studies involving different surgical procedures for diverse surgical pathologies. These factors include obesity, midline incision site, previous abdominal surgery, re-operation through the same incision, wound infection, chronic kidney disease, smoking, prolonged cough, diabetes, jaundice, and urinary obstruction [9,11,46,47].

Clinical picture and presentation

IH can manifest itself in the form of a broad spectrum of disease presentations and progression, ranging from an asymptomatic state up to incarceration with strangulated perforated bowels. Patients with IH could complain of unspecific symptoms such as postprandial fullness, pain, and disfigurement due to large abdominal bulges, which in turn leads to social exclusion [48].

Large IH can be associated with overlying skin changes, dyspnea, insomnia, and limited ability to work. Additionally, in the long term, it can negatively affect the static of the musculoskeletal system and chronic spinal problems [48,49].

The most severe complication which may occur in the natural course of untreated IH is incarceration, which is estimated to affect 6 to 15% of cases of IH. Approximately 4% of patients need surgery to reduce pain, respiratory dysfunction, and discomfort and to prevent sinister complications [48,50].

A recent study of the Danish National Colorectal Cancer Group database included 2466 patients who had surgery for colonic cancer [51]. They assessed the quality of life (QOL) with the development of IH with a median time from colonic cancer resection to QOL assessment of 9.9 years. They found 215 (8.7%) patients developed IH; 156 (72.6%) underwent surgical repair. IH was significantly associated with reduced QOL in the domains of global health, physical functioning, role functioning, emotional functioning, and social functioning, as well as significantly associated with increased symptoms in the scales of pain, dyspnoea, and insomnia. Surgical repair was associated with increased QOL in physical and role-functioning domains [51].

Strategy and options

Given the diverse clinical presentation of IH from asymptomatic to minimally symptomatic condition up to incarceration and the associated comorbidities and intricate surgical field, some would advocate the watchful wait strategy for minimally symptomatic uncomplicated IH [2,52]. However, the scene is quite dynamic, and the hernial defects and sac expand with time [53]. This strategy could be the reason behind the increasing rate of complicated hernia with incarceration and bowel compromise [54]. Incarcerated large IH is among the top 10 causes of emergency laparotomies in the UK. In 2017 it represented 1.3% of all laparotomies according to the 4th NELA report; this incidence has doubled to represent 2.8% of all laparotomies done in the UK in 2020 in the 7th NELA report [55,56].

It has become prominent that surgery has a dramatic change in terms of symptom control and overall QOL. A Swedish study showed that regardless of the surgical technique, all patients reported a quality of life comparable to that of the general population eight weeks after surgery. This improvement has persisted after one year [57]. Moreover, the percentage of patients complaining of symptoms dropped from 81% preoperatively to 18% after surgery [57]. Additionally, surgery led to significant improvements in movement, the feeling of fatigue, and visual analogue scale (VAS) pain score [57]. The same results have been reproduced in a more recent study with improvement in pain, depression and quality of life [58].

On the other hand, there has been evidence of some residual symptoms in many patients after surgical repair of IH [57]. In a recent study, 210 patients were included, and the median follow-up period was 3.2 years [59]. The patients attended the outpatient clinic to collect patient’s reported outcomes (PROs). While 63% of the patients reported experiencing an improvement in the overall condition of their abdominal wall following surgery, an equal percentage reported postoperative symptoms, such as discomfort, pain, and bulging. Furthermore, 20% indicated that the overall status of their abdominal wall remained unchanged, and 17% reported a deterioration compared to their presurgical repair condition. As a result, in retrospect, 10% of the patients would choose not to undergo the operation. This study underscores the significance of effectively managing patient expectations and incorporating PROs in informed consent and decision-making [59].

Surgical techniques and postoperative care

The open surgical technique with retro muscular (sub-lay) mesh placement has been the gold standard and the most popular technique [60]. A meta-analysis of 21 studies that included 5891 procedures showed that sub-lay placement of mesh was associated with the lowest risk for recurrence and surgical site infections (SSIs) [61].

However, with the advances in minimally invasive techniques and training programs, the minimal access IH repair techniques are gaining wide popularity, including laparoscopic and robotic-assisted techniques. These minimally invasive approaches have the advantages of reduced postoperative morbidity, faster recovery, and fewer wound-related complications [62].

In a recent survey, general surgeons in Canada were surveyed to outline their typical surgical approach for a patient with a midline IH and a 10 x 6 cm fascial defect [63]. Among the 483 surgeons surveyed, 74% expressed their preference for conducting an open repair, while 18% favoured laparoscopic repair. Ninety eight percent of the surgeons would opt for using mesh, 73% would undertake primary fascial closure, and 47% would consider a component separation as part of their surgical approach. The mesh was most frequently placed in the retrorectus/ preperitoneal area (48%) and intraperitoneal space (46%). They concluded that although nearly all surgeons conducting IH repairs would opt for permanent mesh, there was considerable diversity in their surgical approaches, choices of mesh placement, techniques for fascial closure and the consideration of component separation.

A meta-analysis that included nine RCTs showed that both open and laparoscopic techniques of IH have similar rates of reoperation and surgical complications and comparable recurrence rates [48]. Recent case series have proved the feasibility of the robotic approach to IH repair with comparable results with laparoscopic surgery [64-66]. However, a tangible clinical benefit does not offset the robotic approach's higher cost and longer operative time [67].

The empirical postoperative care after IH repair would include a period of physical rest in addition to an abdominal binder (AB) or the application of pressure dressing. The former has been meant to avoid early recurrence, and the latter to help prevent seroma formation, reduce pain and improve physical activity. The physical rest after hernia repair was first advised by Bassini after inguinal hernia repair [68]. However, with the evolution of surgical techniques [69], this was challenged through a large case series and RCTs [70,71]. Although the application of AB may reduce pain and improve physical function after major abdominal surgery [72], two dedicated studies did not prove any effect of AB on pain, movement, seroma formation, fatigue, general well-being, or quality of life after ventral and IH repair [73,74].

A recent survey conducted in Germany showed a significant variation in postoperative protocols after IH repair, including postoperative physical rest and the use of AB [49]. Additionally, the same study reviewed six relevant publications on open incisional herniorrhaphy. There was no correlation between the duration of physical rest, SSIs, and the recurrence rate [49].

Surgical outcomes and complications

A broad spectrum of adverse outcomes could be expected in an elderly population with multiple comorbidities after such complex abdominal wall reconstruction procedures. However, SSIs and hernia recurrence are considered direct surgical complications and might need further interventions [75,76].

A study from the USA assessed the effect of these three modifiable comorbidities, obesity, diabetes, and smoking, on wound complications after IH repair [77]. In this study, 3908 patients were included, with 31% having no modifiable comorbidities, 49% having one modifiable comorbidity and 20% having two or more modifiable comorbidities. Compared to individuals without modifiable comorbidities, one modifiable comorbidity or two or more modifiable comorbidities significantly increased the likelihood of SSIs. Nevertheless, only patients with two or more modifiable comorbidities displayed significantly higher odds of surgical site complications necessitating interventions when contrasted with those without modifiable comorbidities and those with just one modifiable comorbidity. Patients who had all three comorbidities experienced a twofold increase in the odds of experiencing any wound-related complications, and obese patients with diabetes exhibited a comparable pattern [77].

Another USA study included 220,629 patients with elective incisional, inguinal, umbilical, or ventral hernia repair from 2011 to 2014. Out of these, 40446 (18.3%) were current smokers. Current smokers experienced an increased likelihood of reoperation, readmission, and death. Furthermore, smokers experienced an increased risk of postoperative complications (including pulmonary, infectious, and wound-related) [78].

Recurrent hernias are considered complex wall hernias, and 20% of all IH repair procedures involve a recurrent hernia [43]. Recurrence rates after IH repair range from 8.7 to 32%, depending on a host of factors, including obesity, use of mesh, setting of repairs, elective versus emergency, and hernial defect size [43,76,79]. The European Hernia Society and Americas Hernia Society guidelines clearly recommend smoking cessation for 4-6 weeks and weight loss to BMI below 35 kg/m2 before elective ventral hernia repair [80].

Cost and burden

The significant complications and recurrence rates of IH management substantially burden healthcare provider facilities [81]. A French study examined the direct costs (related to hospital expenses) and indirect costs (of sick leave) associated with IH repair [82]. The study collected data from 51 public hospitals in France, involving 3239 IH repair procedures. The average overall cost for IH repair in France in 2011 was approximated to be 6451€. This cost varied, with it being 4731€ for unemployed patients and 10107€ for employed patients, whose indirect costs (5376€) were slightly higher than the direct costs. They estimated that a five percent reduction in the incidence of IH following abdominal surgery, achieved through measures like adopting the European Hernia Society Guidelines on abdominal wall incision closure or considering prophylactic mesh augmentation in high-risk patients, could lead to national cost savings of 4 million euros [82].

Another study from the USA projected that between 2012 and 2014, 89258 IH repair surgeries were performed annually, resulting in hospital costs of $6.3 billion [83]. Also, they revealed a strong negative correlation between nonelective IH repair and poorer outcomes, such as postoperative complications, prolonged hospital stay and in-hospital mortality.

Risk prediction and prophylactic measures

From the above, it is evident that every effort should be exercised to help prevent the development of IH. A standardised fascial closure technique after abdominal surgery has reduced the incidence of IH [84]. Additionally, there has been a recent trend toward using prophylactic meshes or suture line reinforcement to prevent IH development after abdominal surgery [85].

In a recent open-label RCT [7], high-risk adult patients aged over 18 years who had undergone a midline laparotomy procedure were followed up for three years. These patients were randomly assigned in a 1:1 ratio to receive either the reinforced tension line (RTL) technique or primary suture only (PSO). The study initially included 124 patients, with 51 from the RTL group and 53 from the PSO group completing the three-year follow-up. The incidence of IH was found to be higher in the PSO group (28.3%) compared to the RTL group (9.8%), and this difference was statistically significant (p = 0.016). Both groups exhibited similar SSI rates, haematoma, seroma, and postoperative pain during the follow-up period.

The STITCH trial was a double-blind, randomised controlled trial that took place across multiple medical centres, specifically within the surgical and gynaecological departments of ten different hospitals in the Netherlands from October 2009 to March 2012 and included a total of 560 patients, which were randomly assigned to either the “large bites” group (comprising 284 patients) or the “small bites” group (consisting of 276 patients) [86]. The groups had a follow-up till August 2013, with 545 (97%) patients completing the follow-up period. Patients in the “small bites” group underwent fascial closures with a greater number of suture stitches, a higher ratio of suture length to wound length, and a longer closure time compared to those with “large bites” closure. After one year of follow-up, it was observed that 57 out of the 277 patients (21%) in the “large bites” group and 35 out of 268 patients (13%) in the “small bites” group had developed IH (p = 0·0220). They concluded that the small bites technique should be the standard closure technique for midline incisions because it prevents IH in midline incisions than the conventional large bites technique.

In contrast, previous trials that examined the impact of techniques involving suture length or modifications in the size of sutures (large bites) did not yield significant results, indicating limited success in demonstrating their effectiveness. A prospective, multicenter, double-blind, parallel-group, randomised controlled superiority trial investigated the influence of suture length on the development of IH during fascia closure [87]. They compared the two suture techniques: one using short stitches (ranging from 5 to 8 mm, spaced every 5 mm) with a USP 2-0 single thread and an HR 26 mm needle, and the other using long stitches (10 mm apart) with a USP 1 double-loop suture and an HR 48 mm needle. Both techniques utilised a suture material based on poly-4-hydroxybutyrate (Monomax®). They compared closure using a short stitch (5 to 8 mm every 5 mm, USP 2-0, single thread HR 26 mm needle) or long stitch technique (10 mm every 10 mm, USP 1, double loop, HR 48 mm needle) with a poly-4-hydroxybutyrate-based suture material (Monomax®). Moreover, they involved 425 patients, who were randomised to either the “short stitch” group (n = 215 patients) or the “long stitch” group (n = 210 patients). After one year of follow-up, it was observed that seven out of 210 patients (3.3%) in the “short stitch” group and 13 out of 204 patients (6.4%) in the “long stitch” group developed IH. However, this difference was not statistically significant (p = 0.173). The initial findings of this trial, observed at the one-year follow-up, indicated a relatively lower incidence of IH in the “short stitch” group. However, this difference did not reach statistical significance.

A more recent prospective, multicenter, single-blinded randomised controlled trial evaluated both the clinical and cost-effectiveness of the Hughes abdominal closure technique compared to the standard mass closure method following colorectal cancer procedures [88]. The study involved 802 adult patients who had undergone surgical resection for colorectal cancer at 28 different surgical sites in the UK. At the one-year follow-up, the incidence of IH, as determined through clinical examination, was 50 cases (14.8%) in the group that used the Hughes abdominal closure technique, compared to 57 cases (17.1%) in the standard mass closure group. However, this difference was not statistically significant (p = 0.4). In the second year, the incidence of IH was 78 cases (28.7%) in the Hughes abdominal closure group and 84 cases (31.8%) in the standard mass closure group, with no statistically significant difference (p = 0.43). Furthermore, the mean incremental cost for patients undergoing the Hughes abdominal closure was £616.45, which also did not reach statistical significance (p = 0.3580). Quality of life did not show a significant difference between the two groups.

Several other trials assessed the effectiveness of prophylactic mesh enhancement after major abdominal procedures. An open-label RCT from Switzerland included 169 patients undergoing elective open abdominal surgery from 2011 to 2014 with a follow-up one year and three years after surgery [89]. They included patients with two or more of the following risk factors: overweight or obesity, diagnosis of neoplastic disease, male sex, or history of a previous laparotomy. Patients were randomly assigned to prophylactic intraperitoneal mesh implantation or standard abdominal closure. Prophylactic intraperitoneal mesh implantation reduced the incidence of IH but increased early postoperative pain and reduced trunk extension. The same results have been reproduced in a more recent retrospective analysis of 309 patients who had open colorectal surgery. Prophylactic mesh closure reduced the incidence of IH but was associated with a higher rate of SSIs [90]. Another study with a five-year follow-up [91] of the PRIMAAT trial [92] included 114 patients; thirty-three in the NO-MESH group (33/58-56.9%) and 34 patients in the MESH group (34/56-60.7%) were evaluated after five years. The cumulative incidence of IHs in the NOMESH group was 32.9% after 24 months and 49.2% after 60 months. No IHs were diagnosed in the MESH group. In the NOMESH group, 21.7% (5/23) underwent re-operation within five years due to an IH.

Aiolfi et al. conducted a systematic review and meta-analysis of RCTs comparing prophylactic mesh reinforcement (PMR) to primary suture closure (PSC) in abdominal surgeries [93]. Their analysis included 14 RCTs involving a total of 2332 patients. Among these patients, 1280 (54.9%) underwent PMR, while 1052 (45.1%) had PSC, and the follow-up period ranged from 12 to 67 months. The results indicated that the incidence of IH was significantly lower in the PMR group compared to the PSC group, with rates of 13.4% and 27.5%, respectively. The estimated pooled relative risk RR for IH in the PMR group compared to the PSC group was 0.38 (p < 0.001). A subgroup analysis, categorised by mesh placement, revealed a reduced risk reduction for various locations: preperitoneal (RR = 0.18; 95% CI 0.04-0.81), intraperitoneal (RR = 0.65; 95% CI 0.48-0.89), retro-muscular (RR = 0.47; 95% CI 0.24-0.92) and on-lay (RR = 0.24; 95% CI 0.12-0.51) compared to PSC. Additionally, the risk of developing seromas was higher in the PMR group (RR = 2.05; p = 0.0008). They concluded that PMR was effective in reducing the risk of IH following elective midline laparotomy in comparison to primary suture closure but appeared to have a higher postoperative risk of developing seromas.

As these preventative prophylactic measures are associated with increased risk of pain, reduced mobility and SSI, there is a need for developing a risk stratification tool to identify those patients with a high risk of IH to justify the utilisation of extra precautions like prophylactic meshes or suture line reinforcement. A recent study assessed preoperative abdominopelvic CT scans’ morphometric, linear, and volumetric measurements to predict IH development after colorectal surgery [94]. The study involved 212 patients, with 106 matched pairs. Out of the 117 features that were measured, 21 of them exhibited the ability to distinguish between patients with IH and those without. Specifically, they identified three morphometric domains on routine preoperative CT imaging that were linked to the presence of IH: the widening of the rectus complex, an increase in visceral volume, and the atrophy of body wall skeletal muscles.

Furthermore, a recent USA study included 29739 patients who had abdominal surgery from 2005 to 2016 [95]. They created eight surgery-specific predictive models for IH with excellent risk discrimination. These included colorectal and vascular surgery. The most prevalent risk factors that raised the probability of developing IH included a history of previous abdominal surgery and smoking. Also, they developed a risk calculator application for preoperative estimation of IH after abdominal surgery.

## Conclusions

IH is a common complication after open and minimal access surgery with a multifactorial pathogenesis. The predisposing factors included inherent and modifiable ones. Elective repair would improve the QOL and prevent the sinister outcomes of emergency IH repair. Accordingly, the watchful wait strategy should be reviewed, and the options should be discussed thoroughly during patients’ counselling. Risk stratification tools for predicting IH would help adopt prophylactic measures like suture line reinforcement or mesh application in high-risk groups.
